# Case Report: Infection-triggered multiple organ dysfunction syndrome as the initial presentation of undiagnosed turner syndrome in an 11-Year-Old girl

**DOI:** 10.3389/fmed.2026.1876783

**Published:** 2026-07-02

**Authors:** Tianqing Zhang, Kailin Yang, Li Luo, Sijie Xiao, Honglan Liu, Junpeng Chen, Xia Zhan, Minghui Wang, Zixin Wu, Yu Zhou, Liangqing Ge

**Affiliations:** 1 Department of Cardiology, Changde Hospital, Xiangya School of Medicine, Central South University (The First People’s Hospital of Changde City), Changde, China; 2 Institute of Basic Research in Traditional Chinese Medicine, Hunan Academy of Chinese Medicine, Changsha, Hunan, China; 3 Department of Ultrasound, Changde Hospital, Xiangya School of Medicine, Central South University (The First people’s Hospital of Changde City), Changde, China; 4 Center for Cardiometabolic Science, University of Louisville, Louisville, KY, United States; 5 Department of Pediatric Ward, Changde Hospital, Xiangya School of Medicine, Central South University (The First people’s Hospital of Changde City), Changde, China; 6 Kentucky Country Day School, Louisville, KY, United States

**Keywords:** case report, multidisciplinary management, multiple organ dysfunction syndrome, turner syndrome, X chromosome deletion

## Abstract

**Background:**

Turner syndrome (TS) is a common sex chromosome disorder typically associated with chronic complications such as short stature, premature ovarian insufficiency, and cardiovascular anomalies. However, acute life-threatening presentations, particularly infection-triggered severe multiple organ dysfunction syndrome (MODS) as the initial manifestation of previously undiagnosed TS in children, are extremely rare.

**Case presentation:**

An 11-year-old girl with previously undiagnosed TS presented with vomiting, altered mental status, fever, diarrhea, and cyanosis. Her condition rapidly progressed to septic shock with heart failure (ejection fraction 35%), acute respiratory distress syndrome, stage III acute kidney injury requiring continuous renal replacement therapy, acute liver injury, and disseminated intravascular coagulation. Genetic testing confirmed a 45, X karyotype with a large Xp22.33–q28 deletion (152.83 Mb). She also exhibited aortic calcification, hypertension, genital hypoplasia, intellectual disability, and characteristic dysmorphic features. Multidisciplinary management with broad-spectrum antibiotics, inotropic support, renal replacement therapy, and antihypertensive treatment led to stabilization. At recent outpatient follow-up, her ejection fraction had recovered to 63%.

**Conclusion:**

This case demonstrates that TS can present as acute, life-threatening MODS in children and highlights the critical need for early genetic diagnosis in girls with unexplained multisystem organ dysfunction and dysmorphic features. Prompt multidisciplinary care can significantly improve prognosis, emphasizing the value of considering TS in such acute critical presentations.

## 1 Introduction

Turner syndrome (TS) is a common sex chromosome disorder affecting phenotypic females, characterized by complete or partial absence of the second sex chromosome. With an incidence of approximately 1 in 2500 live female births, it shows marked clinical heterogeneity across the lifespan, including progressive growth failure due to SHOX haploinsufficiency, premature ovarian insufficiency, and congenital or acquired cardiovascular anomalies—the latter being the leading cause of premature mortality, accounting for more than 40% of excess deaths (1, 2). International guidelines recommend lifelong multidisciplinary monitoring of cardiovascular, metabolic, and neuropsychological complications (2).

While chronic manifestations such as hypertension and aortic dilation are well recognized, life-threatening acute presentations—particularly infection-triggered severe multiple organ dysfunction syndrome (MODS) as the initial manifestation of previously undiagnosed TS in children—remain extremely rare (3).

Here, we report an 11-year-old girl with previously undiagnosed TS (45,X karyotype with a large Xp22.33–q28 deletion spanning 152.83 Mb) who presented with septic shock, heart failure, and severe MODS. This case illustrates the diagnostic challenge of TS manifesting as acute, life-threatening MODS and underscores the critical importance of early genetic diagnosis combined with standardized multidisciplinary management to improve outcomes.

## 2 Case presentation

An 11-year-old girl with previously undiagnosed Turner syndrome presented with infection-triggered severe MODS. She had a history of growth retardation, intellectual disability, and dysmorphic features including simian crease, cubitus valgus, and shield-shaped chest. She was born at term via caesarean section with a birth weight of 2.45 kg and had no family history of genetic disorders.

### 2.1 First admission

The patient, an 11-year-old girl with short stature (height 114 cm, weight 24 kg), was admitted with vomiting and altered mental status for 1 week, fever and diarrhea for 3 days, and cyanosis for 1 day. She had received cephalosporin antibiotics and probiotics locally without improvement and was transferred in septic shock. On admission, vital signs showed temperature 38.8 °C, heart rate 132 beats per minute, respiratory rate 23 breaths per minute, and blood pressure 91/46 mmHg. Physical examination revealed malnutrition, delirium, cyanotic lips, coarse breath sounds, and mild edema. Laboratory results demonstrated leukocytosis, markedly elevated inflammatory markers, severe hepatic injury, stage III acute kidney injury (creatinine 146.8 μmol/L), coagulopathy consistent with disseminated intravascular coagulation, and myocardial injury with an ejection fraction of 35%. Blood and stool cultures were negative for bacterial and fungal growth. Her condition rapidly progressed to MODS involving septic shock with heart failure (ejection fraction 35%), acute respiratory distress syndrome (PaO_2_/FiO_2_ 21), and disseminated intravascular coagulation (Table 1). Chest radiography revealed bilateral pulmonary infiltrates and cardiomegaly.

**TABLE 1:** Key organ function details.

Organ/System	Clinical manifestations	Key indicators	Relevance to Turner syndrome
Circulation	Shock	LDH 4875 U/L; CK 13302 U/L; CK-MB 140.5 U/L; Myo 6434.75 U/L; cTnI 1.982 ng/mL; pro-BNP > 35000 pg/mL	May be exacerbated by underlying TS-associated congenital cardiovascular anomalies, aortopathy, and hypertension, increasing susceptibility to myocardial injury and cardiogenic shock during severe infection.
Respiratory	ARDS	PaO_2_/FiO_2_ = 21; pO_2_ 69.0 mmHg; pCO_2_ 29.9 mmHg	Secondary to heart failure and cardiogenic pulmonary oedema, which may be worsened by TS-associated cardiac dysfunction.
Kidney	AKI III phase, need CRRT	Cr 146.8 μmol/L	Increased vulnerability to hypoperfusion and AKI in the setting of TS-related cardiovascular compromise and septic shock.
Liver	Abnormal Liver Function	ALT 1094 U/L; Bilirubin 37.78 μmol/L	May be exacerbated by TS-related baseline cardiovascular and metabolic stress during severe systemic insult.
Blood coagulation	DIC	PT 29.30 s; INR 2.91; APTT 35.40 s; D-dimer 8.83 mg/L; FDP 75.49 μg/mL; AT3 34.5%	Secondary to septic shock and multi-organ failure; hepatic synthetic dysfunction is common in critical illness.
Internal environment	Severe electrolyte and acid-base disturbance	K^+^ 2.70 mmol/L; Na^+^ 126.0 mmol/L; Ca^2+^ 0.92 mmol/L; lactate 5.30 mmol/L; plasma renin > 500 pg/mL, aldosterone 27.936 ng/dL (supine), angiotensin II 76.870 pg/mL	Exacerbated by AKI and renin–angiotensin–aldosterone system (RAAS) activation, which is frequently observed in TS patients with hypertension.

She was treated in the intensive care unit with broad-spectrum antibiotics (meropenem and ceftriaxone), hemodynamic support (dopamine 1.65 μg/kg/min and dobutamine 5 μg/kg/min), and continuous renal replacement therapy. Her cardiac function improved (ejection fraction rising to 45%) prior to discharge, although blood pressure remained unstable.

### 2.2 Second admission and diagnosis

Two months later, the patient was readmitted with a 10-day history of intermittent vomiting, dizziness, and dyspnea following a recent Influenza A infection. She had not attended regular follow-up. Vital signs showed temperature 37.1 °C, heart rate 106 beats per minute, respiratory rate 40 breaths per minute, and blood pressure 159/111 mmHg. Physical examination revealed altered mental status, pallor, cyanotic lips, tachypnea, and coarse crackles in the lungs. She was found to have rhinovirus infection, heart failure (BNP 6013 pg/mL, ejection fraction 41%), mild mitral and tricuspid regurgitation, mild pericardial effusion, coagulopathy, and hypocalcemia. Computed tomography angiography demonstrated calcification of the descending and abdominal aorta with possible mural thrombus and cardiomegaly (Figure 1). Pelvic ultrasound confirmed an infantile uterus, bilateral ovarian agenesis, and breast hypoplasia (Figure 2). Genetic testing confirmed the diagnosis of Turner syndrome: whole-exome sequencing identified a large 152.83 Mb deletion in the Xp22.33–q28 region, and G-banding karyotype analysis revealed a 45,X karyotype (Figure 3).

**FIGURE 1:**
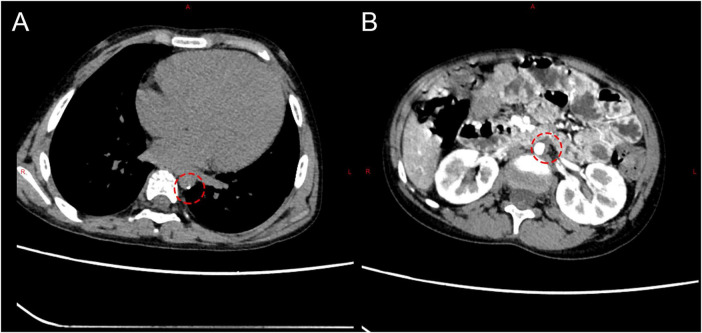
CT angiography demonstrating calcification of the descending **(A)** and abdominal aorta **(B)** with possible mural thrombus (red circles) and cardiomegaly.

**FIGURE 2:**
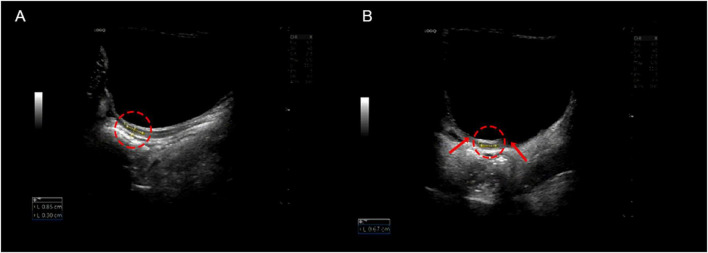
Pelvic color Doppler ultrasound [Longitudinal **(A)** and transverse **(B)** views demonstrating a rudimentary uterus (outlined in red) and bilateral streak gonads (arrows; no ovarian tissue visible in the adnexa)].

**FIGURE 3:**
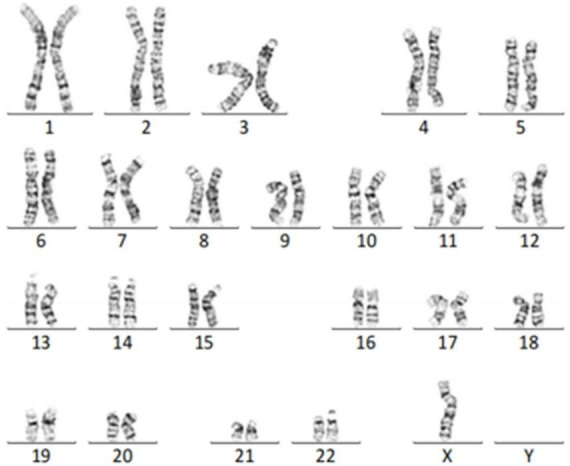
G-banding karyotype analysis revealing a 45,X karyotype, confirming Turner syndrome.

Multidisciplinary management was continued with anti-infective therapy, combined antihypertensives (amlodipine plus valsartan), and heart-failure treatment. During the outpatient follow-up 1 month later, the ejection fraction had recovered to 63%, and the patient’s condition remained stable.

## 3 Discussion

This case demonstrates the typical phenotypes of TS, including growth retardation [height 114 cm due to SHOX haploinsufficiency (4)], ovarian dysgenesis, and cardiovascular abnormalities (hypertension, and heart failure). Cardiac defects occur in 35%–50% of TS cases overall, but symptomatic heart failure and infection-triggered severe MODS are uncommon in children. However, infection-triggered acute MODS may have been amplified by TS-associated cardiovascular compromise, hypertension, and possible immune dysregulation, although a direct causal relationship cannot be established. Recent studies have described immune alterations in TS, including reduced T-cell subsets, altered CD4:CD8 ratios, and hypogammaglobulinemia, which may increase susceptibility to severe infections (5, 6). Furthermore, this patient has a non-mosaic 45, X karyotype with complete monosomy of the X chromosome. Such profound X-chromosome haploinsufficiency may contribute to more severe immune dysregulation and a higher risk of acute decompensation compared with mosaic TS or cases with smaller structural abnormalities. The patient also exhibited the uncommon combination of simian crease and intellectual disability [due to X-linked gene dosage effects (3, 7)], which highlights the need for neuropsychological screening in children with idiopathic short stature who present with acute critical illness (3, 7).

This case also illustrates the two key challenges in diagnosing TS and their corresponding solutions. The first challenge is the differential diagnosis of multisystem organ dysfunction: in children with unexplained MODS accompanied by dysmorphic features, Turner syndrome should be considered early in the differential diagnosis, particularly in girls with short stature or genital hypoplasia (8). The second challenge concerns the timing of genetic testing: whole-exome sequencing (WES) confirmed the diagnosis of TS within 3 weeks of admission, enabling targeted management. According to the 2024 international guidelines, peripheral blood karyotype analysis is recommended as the first-line method (requiring analysis of at least 30 metaphase cells to detect mosaicism), while complex cases may be supplemented with WES or copy number variation sequencing (CNV-seq) (7). In this case, WES played a critical role in ruling out alternative diagnoses (such as Fabry disease and mitochondrial disorders) and confirming X monosomy.

Multidisciplinary management of this patient highlighted the critical role of integrating acute and chronic care. In the acute phase, early broad-spectrum antibiotics (meropenem) and hemodynamic support (dopamine) effectively addressed sepsis and MODS. Combined antihypertensive therapy along with neurohormonal antagonists improved the ejection fraction (from 35% to 45% at first discharge, with a transient decline to 41% during the second admission likely related to rhinovirus infection, followed by recovery to 63% at recent outpatient follow-up) and controlled hypertension (8). This case uniquely demonstrates that timely MDT intervention can reverse life-threatening acute decompensation in undiagnosed TS, even when MODS is the first presentation.

This study has several limitations. First, as a single case report, the findings cannot be generalized. Second, although the clinical picture was consistent with infection-triggered MODS, blood and stool cultures were negative during the first admission, and only rhinovirus was detected during the second admission; thus, the exact causative pathogen remains unconfirmed. Non-infectious causes of acute myocardial injury (e.g., stress cardiomyopathy or viral myocarditis) cannot be entirely excluded. Third, follow-up duration is relatively short.

## Data Availability

The original contributions presented in this study are included in this article/supplementary material, further inquiries can be directed to the corresponding authors.
